# Feasibility, usability, and validity assessment of a novel plug-and-play virtual endoscopy simulator

**DOI:** 10.1007/s00464-025-12396-8

**Published:** 2025-12-04

**Authors:** Philipp Hohlstein, Marcus Hollenbach, Oscar Cahyadi, Maximilian Beck, Miriam Bittel, Stavros Dimitriadis, Jakob Garbe, Mark E. Geissler, Myriam W. Heilani, Yvonne Huber, Alexander Koch, Leah Kruse, Antonia Mondorf, Sophie Schlosser-Hupf, Jonas J. Staudacher, Lukas Welsch, Thomas von Hahn, Florian A. Michael, Karim Hamesch

**Affiliations:** 1 Department of Gastroenterology, Metabolic Disorders and Intensive Care Medicine (Department of Medicine III), University Hospital RWTH Aachen, Aachen, Germany; 2 Junge Gastroenterologie (JuGa) - German Young Gastroenterology Study Group, DGVS, Berlin, Germany; 3 Interdisciplinary Endoscopy, University Hospital RWTH Aachen, Pauwelsstraße 30, 52074 Aachen, Germany; 4 Department of Gastroenterology, Endocrinology, Metabolism and Clinical Infectiology, University Hospital of Giessen and Marburg Campus Marburg, Marburg, Germany; 5 Department of Gastroenterology, Sankt Josef-Hospital, Ruhr-University Bochum, Bochum, Germany; 6 Agaplesion Bethesda Krankenhaus Bergedorf, Hamburg, Germany; 7 Department of Medicine 1, Friedrich-Alexander-University Erlangen-Nürnberg, Erlangen, Germany; 8 Unit of Hybrid Interventional Endoscopy, Department of Gastroenterology, Mediterraneo Hospital, Glifada, Greece; 9 Clinic for Internal Medicine I, University Hospital Halle, Halle, Germany; 10 Else Kroener Fresenius Center for Digital Health, Faculty of Medicine and University Hospital Carl Gustav Carus, TUD Dresden University of Technology, Dresden, Germany; 11 Medical Clinic 1, University Hospital Goethe, University Frankfurt, Frankfurt, Germany; 12 Department of Medicine I, University Medical Center of the Johannes Gutenberg University Mainz, Mainz, Germany; 13 Department of Internal Medicine I, University Hospital Regensburg, Regensburg, Germany; 14 Department of Gastroenterology, Rheumatology and Infectiology, Charité Universitätsmedizin Berlin, Berlin, Germany; 15 Berlin Institute of Health at Charité, Berlin, Germany; 16 Department of Gastroenterology, Diabetology and Infectiology, Klinikum Hanau gGmbH, Hanau, Germany; 17 Gastroenterology, Hepatology and Interventional Endoscopy, Asklepios Hospital Barmbek, Hamburg, Germany

**Keywords:** Simulation, Simulation-based training, Quality management, Tip control, Endoscopy, Training

## Abstract

**Background:**

A virtual endoscopy simulator with a 3D-printed endoscope handle (“Endonix®”) that can be used without dedicated equipment (“plug and play”) to train endoscope handling has yet not been tested. We prospectively evaluated the simulator’s validity, feasibility, and usability in novices, beginners, and experienced endoscopists.

**Methods:**

Delegates at two congresses (DGVS and ENDOCLUBNORD, both 2024 in Germany) were invited. 310 complete datasets (146 DGVS, 164 ENDOCLUBNORD) were analyzed. Each participant performed two different simulation modules and completed a survey comprising questions on endoscopy experience, simulator evaluation, as well as the System Usability Scale (SUS) and the National Aeronautics and Space Administration Task Load Index (NASA-TLX).

**Results:**

The simulator demonstrated good face validity, but only merely sufficient content validity as 67.8% of all participants favored its implementation into endoscopy training. Construct validity was also favorable as experienced endoscopists completed both modules faster than novices or beginners (*P* < 0.001). In line, criterion validity was given as performance correlated with self-assessed experience. The SUS yielded 75 out of 100 points (corresponding to good usability) with no discernible difference between novices, beginners, and experienced endoscopists. The NASA-TLX, ranging from 0 to 600 points, reflecting level of exhaustion, exhibited a higher score in novices and beginners than experienced endoscopists (285 vs. 210 vs. 225 points, respectively, *P* < 0.001). Only 16.1% of participants had access to a simulator at their respective institutions, while 79.4% wished for the incorporation of simulators into endoscopy training.

**Conclusion:**

The first validation of a novel virtual simulator demonstrated its feasibility and usability, with mostly sufficient validity. Many participants favored its implementation in endoscopy training, particularly for novice and beginner endoscopists, who were identified as the most suitable target learners.

**Graphical Abstract:**

**Figure:**
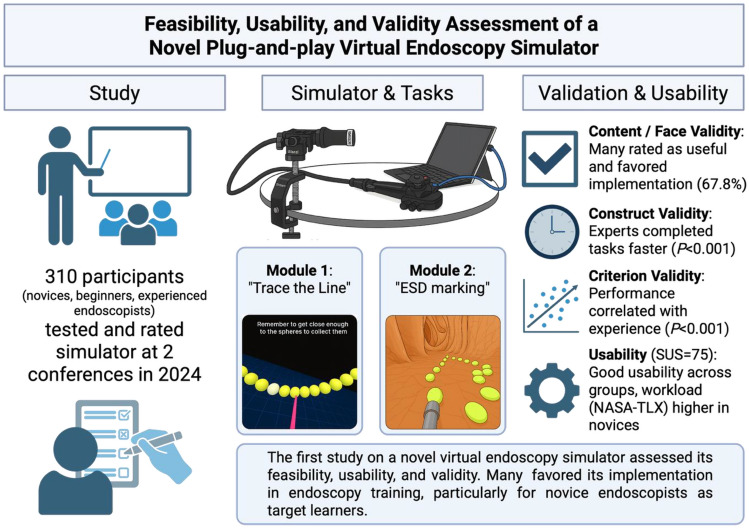
Created in BioRender. Hamesch, K. (2025) https://BioRender.com/q0x2gxz

**Supplementary Information:**

The online version contains supplementary material available at 10.1007/s00464-025-12396-8.

Ensuring high-quality endoscopy is a priority and literature on its implementation is increasing [1–3]. In this regard, the role of simulators is evolving, while a step-by-step approach with initial observation, followed by simulator-based endoscopy focused on scope manipulation and tip control training prior to hands-on endoscopy on patients is recommended [4–8]. By teaching technical, cognitive, and integrative skills prior to endoscopy in patients, the risk for patients is expected to be minimized [9, 10]. In addition, simulators might reduce learning time and number of cases required to learn a new procedure [11]. Several studies indicate that trainees do not reliably reach competency during the dedicated training period [12, 13]. To close this gap, the use of simulators might be useful [4–6, 14, 15]. The implementation of simulators can help to equalize prerequisites and to increase the learning curve. Ideally, simulators reduce the number of necessary procedure numbers to obtain competency [14]. Moreover, simulators offer the possibility to train procedures in a controlled setting without pressure of time or fear of complications compared to the direct training involving patients [16].

Endoscopy simulators can be classified into three different groups: i) mechanical simulators, ii) animal simulators (ex vivo and in vivo), and iii) virtual simulators [17]. Mechanical and animal simulators offer realistic scenarios with haptic feedback comparable to endoscopy in patients. However, both simulator groups deliver only limited and redundant training scenarios, and their availability and storage are complex. Moreover, animal models face ethical and hygiene challenges and are expensive as dedicated endoscopes are needed. While ex vivo tissue samples require specific handling, in vivo animals necessitate on-site care facilities and veterinary staff. Virtual simulators circumvent challenges regarding reproducibility and availability, while possibly being less realistic and lacking haptic feedback. However, favorable features are a wide range of standardized virtual simulations that can be constantly updated and adapted to new technical developments with the option of educational feedback (without an on-site supervisor) as well as a performance measurement and user comparability [18].

This study prospectively evaluated a novel virtual simulator for the first time in an exploratory manner. The aim was to evaluate its validity, feasibility, and usability among novice, beginner, and experienced endoscopists, as well as to determine the most suitable target learners for this device.

## Materials and methods

### Study participants

All delegates at two medical congresses (Visceral Medicine 2024 held by the German Association for Gastroenterology, Digestive and Metabolic Diseases (DGVS) in Leipzig, Germany and ENDOCLUBNORD 2024 in Hamburg, Germany) were invited to anonymously participate in the study. Delegates included physicians, nurses, medical students, and industry representatives. Participants were divided into groups according to their self-assessed skill reported in the survey. To ensure internal plausibility and consistency of these self-assigned categories, we subsequently cross-validated the groups post hoc using objective parameters of endoscopy experience and occupation: Novices were defined as participants without any endoscopic experience (no esophagogastroduodenoscopies (EGD) or colonoscopies performed), while also including other medical personnel or medical students as comparators (due to lacking experience in endoscope handling). Beginners were defined by their self-assessment and an experience of greater than zero endoscopies (EGD or colonoscopies). Experienced endoscopists were defined by their self-assessment (independent endoscopic practice).

### Virtual simulator and survey

**Fig. 1:**
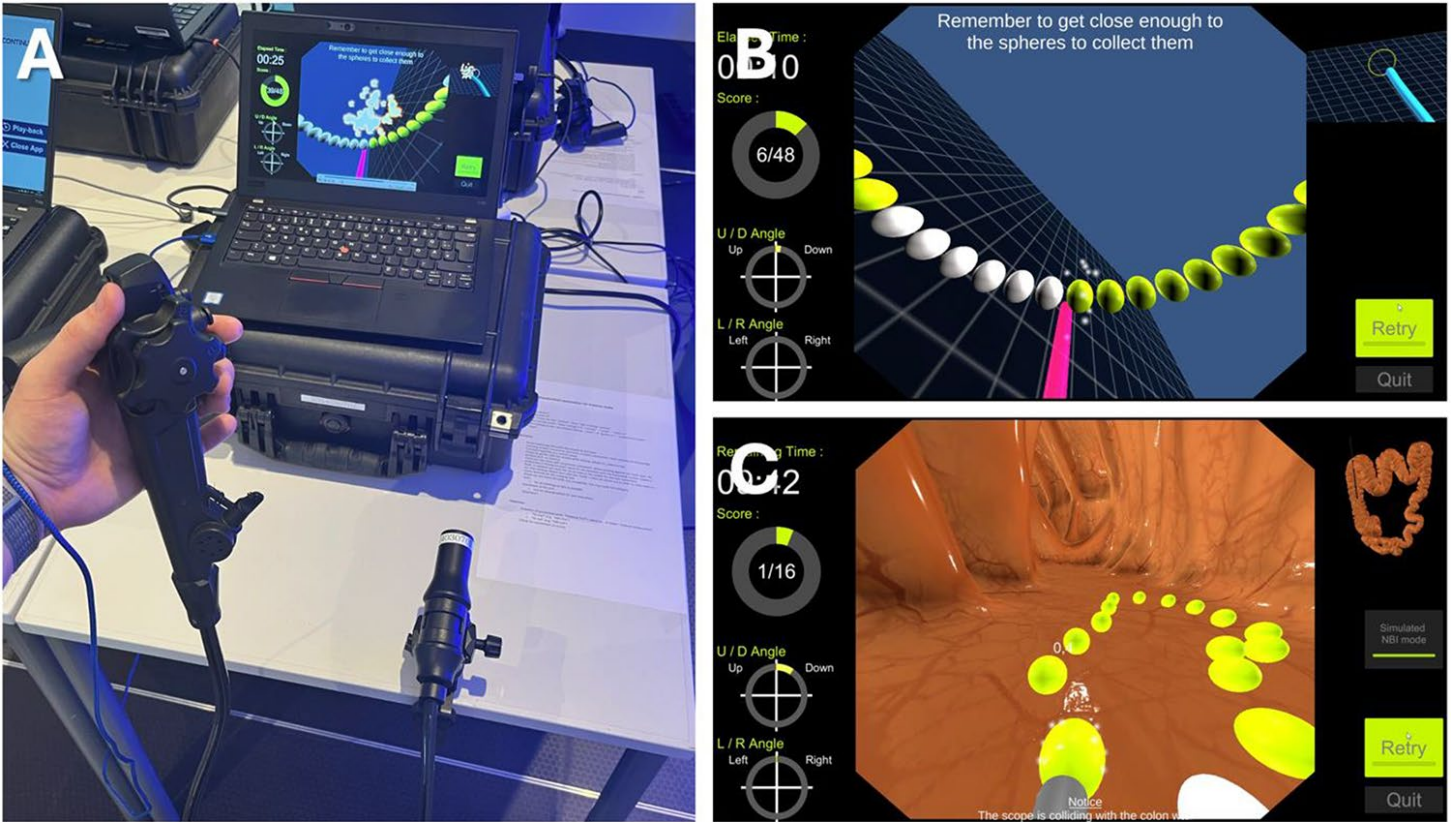
Photo of the simulator and the simulation. **A** Endoscope handle (controller) plugged into a laptop with the distal end of the shaft fixed to the table. Screenshots of the virtual simulation of “Trace the Line” **B** and “ESD marking” **C**

All Endonix® simulators in this study along with the software were kindly provided by Olympus Germany as part of an unrestricted, unconditional loan to the corresponding author. There was no financial grant or influence on the study design, data collection, analysis or manuscript drafting. The Endonix® virtual endoscopy simulator consists of a 3D-printed endoscope handle that can be plugged into a computer. It can be used immediately without the need for any other equipment (“plug and play”) after starting the associated software (Olympus, Tokyo, Japan). The simulation has the style of a video game. The handle resembles a regular endoscope handle with realistic angulation knobs for the Up/Down (U/D) and Right/Left (R/L) control. A dummy instrument can be used to advance instruments through the working channel during simulation. All other buttons on the endoscope handle are non-functional. Furthermore, the endoscope shaft, which is fixed to a terminal, can be rotated, pushed and pulled similar to a regular scope (Fig. 1A). Upon hitting a stop when pushing or pulling the shaft, the endoscope keeps advancing or retracting in the virtual simulation. These controls simulate real-life regular endoscopic navigation. The term “tip control” refers to the skill to precisely manipulate the tip of the endoscope to navigate it through the gastrointestinal tract or to perform procedures, such as resections. The software offers basic as well as procedure training, with exercises ranging from basic modules for learning scope manipulation (i.e., “Trace the Line,” “Fix the Center”) to more advanced procedure training (i.e., “Polyp Detection,” “Biopsy,” “ESD marking,” and “Observation”).

After standardized instruction, all participants were offered to complete the same two training modules (“Trace the Line” and “ESD marking”). Each module starts with an instruction and tutorial screen on the learning objective and task to be performed, including a short video snippet of the exercise. After hitting the “Play” button, the user is shown a virtual endoscopy screen, including elapsed time, score, U/D and R/L angles of the scope on the left side, as well es a small bird’s eye overview of the scene on the right side of the screen. After a “Ready,” then “Go!” instruction, the user can start to maneuver the scope through the virtual endoscopy surrounding. After collecting the last sphere, the user is presented with a result view showing total time and achieved score. In the first module, the objective was to collect 48 spheres arranged in a vertical circle by touching them with the tip of a virtual instrument controlled by the 3D-printed endoscope handle in a regular manner (“Trace the Line,” Fig. 1B, Suppl. Video 1). The exercise is designed to train tip control by controlling the U/D and R/L angulation knobs in conjunction with twist and shaft movements. The vertical circle was chosen after internal pilot testing to require those movements and challenge dexterity across all angulation axes while maintaining visual simplicity for quick reproducibility in a time-constrained environment. The second module (“ESD marking”) required the user to collect 16 spheres by maneuvering the endoscope along an irregularly shaped circle on a colon wall and marking them with the tip of a virtual instrument, mimicking the marking around a lesion to be resected by endoscopic submucosal dissection (ESD) (Fig. 1C, Suppl. Video 2). Both exercises had a time limit of 10 min, after which the exercise ended with the result view.

Prior to and following simulator usage, each participant was asked to complete an anonymous survey utilizing single choice, fill-in, and Likert-style questions ranging from 1 (Strongly disagree) to 5 (Strongly agree). Surveys were created using the online service PaperSurvey.io (Mygtukynas, Vilnius, Lithuania). The survey consisted of questions on demographic data and occupation (age, gender, country, and current profession), endoscopy experience (years of experience, numbers of endoscopies performed, and self-assessment of skills), simulator usage and implementation in general, as well as questions regarding face and content validity of the Endonix® simulator using Likert-style questions as well as validated scoring systems, such as the System Usability Scale (SUS), which evaluates the general usability of the system and the first part of the National Aeronautics and Space Administration Task Load Index (NASA-TLX). The NASA-TLX is a subjective, multidimensional assessment tool that measures perceived workload of a task, system, or team’s effectiveness or other aspect of performance (task loading). It evaluates six subdomains: mental, physical, and temporal demand, as well as performance, effort, and frustration. In this study, the NASA-TLX was applied in its unweighted form, yielding a total score ranging from 0 to 600, with higher scores reflecting greater perceived workload [19–21].

### Validity criteria definitions

Validity assesses whether the simulator effectively teaches or evaluates what it is designed to measure. The four types of validity encompass 1) content validity, 2) face validity, 3) construct validity, and 4) criterion validity [22–24].

### Content and face validity

While content validity [25] describes whether a test (i.e., the simulator) adequately represents the construct it aims to measure, face validity [26] evaluates whether the simulator was appropriate for its intended purpose. Therefore, the research questions posed were as follows: “*Does the simulator appear realistic?*”* and *“*Should it be implemented in endoscopy training curricula?*” After having tested the virtual simulator, i.e., having performed the two exercises “Trace the Line” and “ESD marking” as described above, participants were asked to complete a survey on the different dimensions of validity. However, the interpretation of these results, particularly regarding content validity, should predominantly rely on evaluations provided by physicians with established endoscopic experience.

### Construct validity

Construct validity [27] describes how well a test, i.e., the simulator, measures the concept it is designed to measure. Hence, we examined whether experienced endoscopists outperformed the other groups in the virtual simulation.

### Criterion validity

Criterion validity [28] investigates how well a measurement (i.e., the time to completion in the virtual simulation) corresponds to other established and valid measures of the same concept. Therefore, we examined how strong time to completion of the “Trace the Line” and “ESD marking” exercises correlated with established measures of endoscopy experience (i.e., years of experience and number of endoscopies performed).

### Ethical approval

Ethical approval was waived after review of the study protocol by the local ethics committee due to the anonymous nature of the study (EK 24–352, ethics committee of the University Hospital RWTH Aachen, RWTH Aachen University, Aachen, Germany) and was performed according to the ethical standards demanded in the 1964 Declaration of Helsinki as well as all other applicable security and ethics guidelines.

### Statistical analysis

Statistical analysis and data visualization was performed using Python (Version 3.11.0, Python Software Foundation, Beaverton, USA). Data are presented as median and interquartile range (IQR) due to the skewed distribution of most parameters. Continuous data are visualized as violin plots with an embedded box-and-whisker plot with a white dot indicating the median and a surrounding kernel density estimate to show the distribution. The Kruskal–Wallis test followed by a Dunn’s multiple comparison test was used for unpaired samples with more than two groups. A *P* value of ≤ 0.05 was considered statistically significant.

## Results

### Study participants

**Fig. 2:**
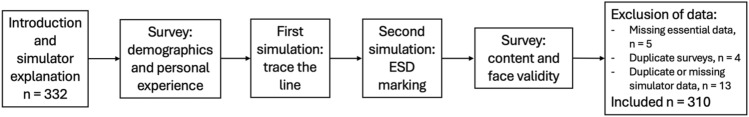
Study design

Of 332 participants, 310 complete datasets (*n* = 146 DGVS, *n* = 164 ENDOCLUBNORD) were analyzed. 22 participants were excluded due to missing essential data in the questionnaire (*n* = 5), duplicate surveys (*n* = 4), and duplicate or missing simulator completion data (*n* = 13) (Fig. 2). Of the 310 participants, 114 were classified as novices, 49 as beginners, and 147 as experienced endoscopists. In proportion, more women with a lower level of experience and more men with a higher level of experience participated (percentage of females: novices 69.3%, beginners 55.1%, experienced 40.1%). Among participant groups, novices had the highest proportion of nurses and students (52.6%), beginners had the highest proportion of resident physicians (59.2%), and experienced endoscopists the highest proportion of specialists (Gastroenterology and Surgery, 87.1%). These data demonstrate adequate segmentation of the three groups. Most of the participants currently practice in the European Union (EU), with the vast majority practicing in either Germany, Austria, or Switzerland (91.0%). Endoscopy experience increased from novice to experienced endoscopists (median of 0, 1, and 7 years, respectively), along with the overall performed EGDs (0, 25, and 2500, respectively) and overall performed colonoscopies (0, 25, and 1750, respectively). Only experienced endoscopists reported performing ESD with 18.4% of this group. Regarding current simulator implementation and training curricula, 16.1% of participants reported having access to a simulator at their institution, while 21.0% of participants reported that their institution had a structured curriculum for endoscopy training. 97.7% of participants successfully completed the “Trace the Line” module, while 94.2% completed the “ESD marking” module. Each participant completed at least one of the exercises to be included in the analysis (Fig. 1 and Table 1).

**Table 1:** Baseline characteristics of study participants

	Novice	Beginner	Experienced
n	114	49	147
Congress (DGVS / ENDOCL (BNORD))	66 / 48	24 / 25	56 / 91
Age, years (IQR)	31 (9)	33 (8)	41 (17.5)
Female gender, n (%)	79 (69.3)	27 (55.1)	59 (40.1)
Profession (nurse or student/resident/specialist IM/specialist GI or surgery), *n* (%)	60 / 29 / 1 / 6 (52.6 / 25.4 / 0.9 / 5.3)	4 / 29 / 10 / 4 (8.2 / 59.2 / 20.4 / 8.2)	5 / 8 / 36 / 98 (3.4 / 5.4 / 24.5 / 62.6)
Country of current practice (DE or AT or CH/other EU/non-EU or missing), *n* (%)	107 / 3 / 4 (93.9 / 2.6 / 3.5)	41 / 7 / 1 (83.7 / 14.3 / 2.0)	134 / 10 / 3 (91.2 / 6.8 / 2.1)
Endoscopy experience, years (IQR)	0 (0)	0 (1)	7 (17)
EGD performed, *n* (IQR)	0 (0)	25 (135)	2500 (9200)
Colonoscopies performed, *n* (IQR)	0 (0)	25 (0)	1750 (9550)
Performs ESD, *n* (%)	0 (0)	0 (0)	27 (18.4)
Institution owns simulator, *n* (%)	20 (17.5)	12 (24.5)	18 (12.2)
Institution with structured curriculum, *n* (%)	21 (18.4)	12 (24.5)	32 (21.8)
Completed module “Trace The Line,” *n* (%)	113 (99.1)	46 (93.9)	144 (98.0)
Completed module “ESD marking,” *n* (%)	104 (91.2)	47 (95.9)	141 (95.9)

The median and interquartile range (IQR, in parentheses) are given unless indicated otherwise. *GI* gastrointestinal, *EGD* esophagogastroduodenoscopy, *ESD* endoscopic submucosal dissection

### Pre-simulator survey

**Fig. 3:**
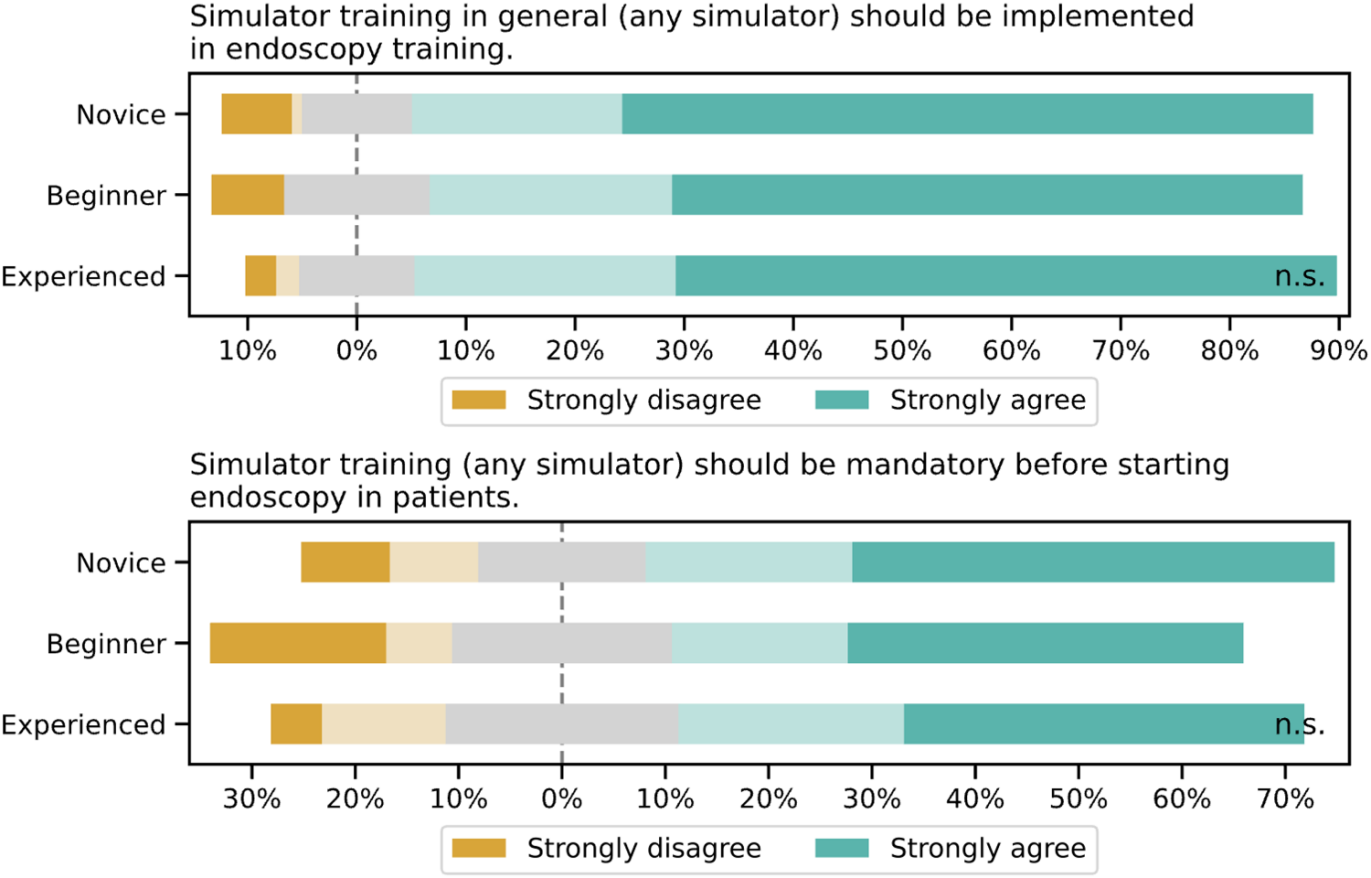
Perception of simulator training in endoscopy among different experience levels (Novice, Beginner, Experienced), assessed using 5-point Likert scales (1 = strongly disagree, 5 = strongly agree). Top: General support for implementing simulator training in endoscopy curricula. Bottom: Opinion on mandatory simulator training prior to performing endoscopy on patients. Using the Kruskal–Wallis test, no significant differences between the groups were detected for both questions

Prior to using and evaluating the simulator, participants were asked two questions to assess their desire to implement simulators in endoscopy training independent of the virtual simulator being tested. Participants supported the use of simulators in endoscopy training (83.1% across all groups) without significant differences between the groups. Of all participants, 61.9% agreed or strongly agreed to mandatory simulator training on any simulator prior to performing endoscopy on patients, again without significant inter-group differences (Fig. 3).

### Content and face validity

**Fig. 4:**
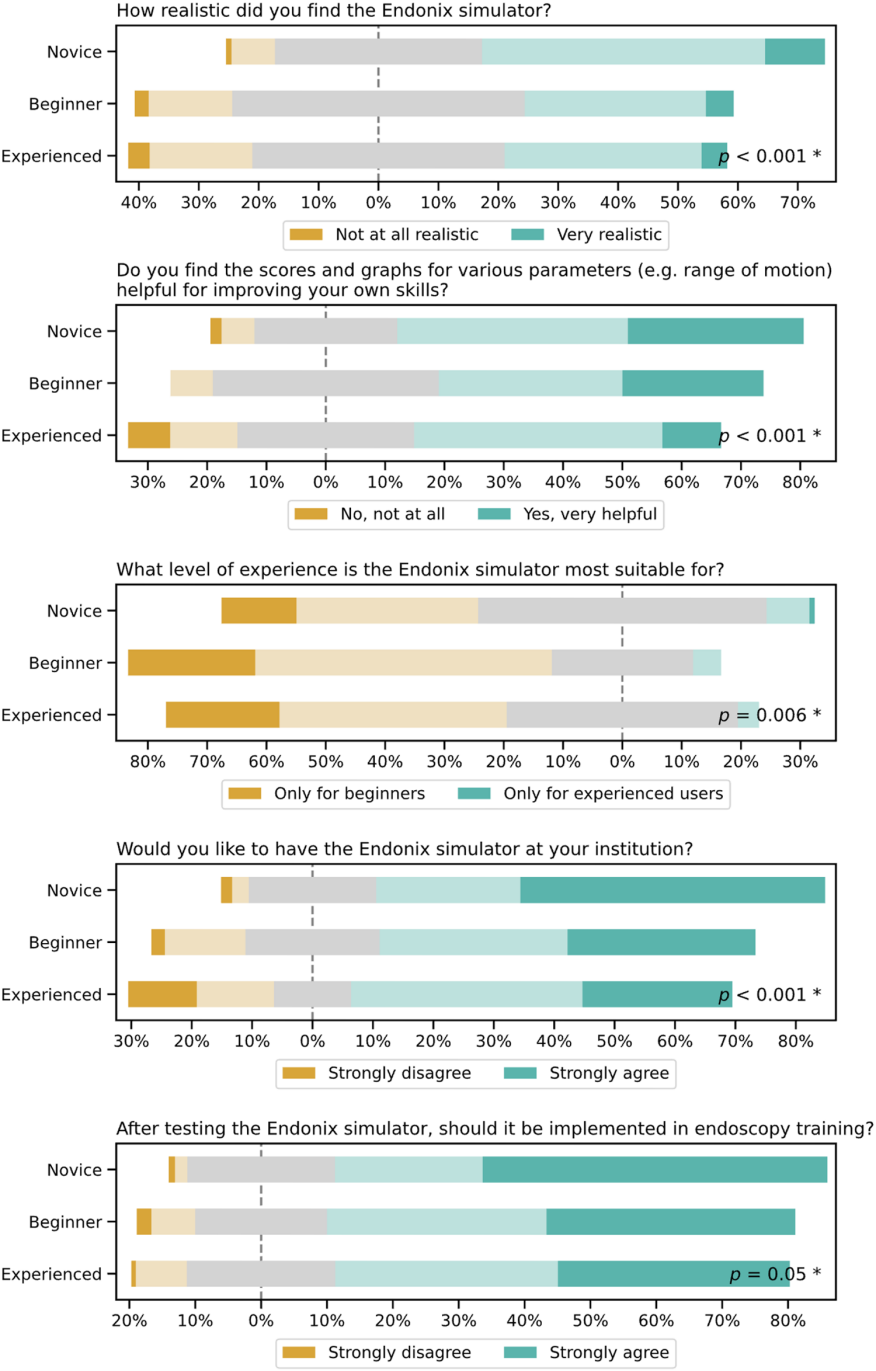
Evaluation of content and face validity of the virtual Endonix simulator on a five-point Likert scales (description below plots) assessing realism, usefulness of feedback metrics, target group, interest in institutional availability, and integration into training depending on varying endoscopy experience level (Novice, Beginner, Experienced). *Significance (*p* < 0.05) between groups was assessed using the Kruskal–Wallis test

Concerning content validity, participants were asked how realistic they found the simulator. Here, 57.3% of novices, but only 34.9% of beginners and 37.2% of experienced endoscopists agreed or strongly agreed (*P* < 0.001). To assess face validity, participants were questioned whether they considered installing the simulator at their institution or if they thought the simulator should be implemented in endoscopy training. Among novices, 74.4% expressed the desire to own the simulator at their institution, compared to 62.2% of beginners and 63.1% of experienced endoscopists (*P* < 0.001). The desire to use the simulator in endoscopy training was stronger among novices than among beginners and experienced endoscopists (74.4%, 71.1%, and 69.0%, respectively, *P* = 0.05). Across participant groups, 58.4% found the simulator’s scores and graphs helpful in improving their own skills, with a slightly higher proportion of novices agreeing (*P* < 0.001). Most participants (54.1%) found the simulator to be useful for less-experienced endoscopists (Fig. 4).

**Fig. 5:**
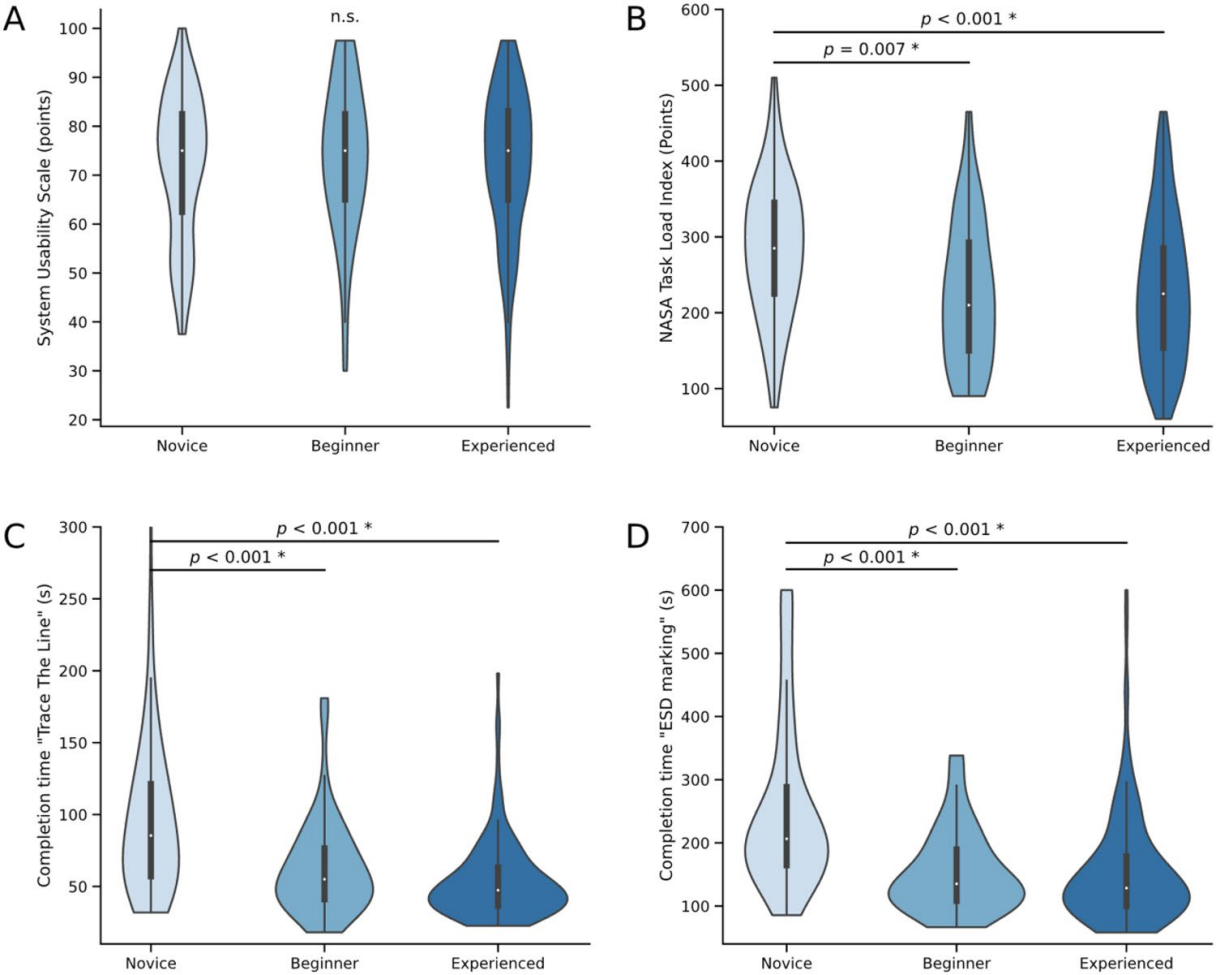
Usability and construct validity of the virtual Endonix simulator. **A** System usability scale (SUS) and **B** NASA Task Load Index (NASA-TLX) across endoscopy experience levels. Time to completion of the exercise “Trace the Line”** C** and “ESD marking” **D**. *Significance (*P* < 0.05) between groups was assessed using the Kruskal–Wallis test, followed by Dunn’s multiple comparison test

The SUS was used to assess the general usability of the virtual simulator. The median SUS score was 75 points, corresponding to good usability (Fig. 5A) without significant inter-group differences. In addition, the SUS items were analyzed: novices and beginners reported a greater willingness to use the system more frequently than experienced endoscopists (*P* < 0.001). Furthermore, novices were more likely than beginners and experienced endoscopists to require assistance from a technician or needed training before using the system (*P* = 0.013 and *P* = 0.008, respectively). Finally, experienced endoscopists found the various functions of this system to be less well integrated than novices and beginners (*P* = 0.034, Figure S2).

The NASA-TLX was used to assess task load intensity. The total task load was higher in novices compared to beginners (median 285 vs. 210 points, *P* = 0.007), as well as compared to experienced endoscopists (median 285 vs. 225 points, *P* < 0.001, Fig. 5B). Novices showed higher task load than beginners and experienced endoscopists on all items of the NASA-TLX including mental, physical, and temporal demands, as well as performance, effort and frustration. In addition, beginners had higher median NASA-TLX sub-item ratings than experienced endoscopists only for mental demand and performance. Interestingly, frustration was slightly higher in experienced than in beginner endoscopists (*P* = 0.002, Figure S3).

### Construct validity

Time to completion of the “Trace the Line” and “ESD marking” modules was longer for novices compared to beginners (85.4 s vs. 55.0 s, *P* < 0.001) and compared to experienced endoscopists (85.4 s vs. 47.4 s, *P* < 0.001). No significant difference was found between beginners and experienced endoscopists (Fig. 5C, D). Additionally, a detailed comparison of occupational backgrounds among novice endoscopists can be found in the supplement (Suppl. Figures S5–7, Suppl. Table S1).

### Criterion validity

For the “Trace the Line” module, we detected moderate to strong negative correlations of completion time with years of endoscopy experience (Spearman’s r −0.26, *P* < 0.001), with number of EGDs performed (Spearman’s r −0.39, *P* < 0.001), and with the number of colonoscopies performed (Spearman’s r −0.39, *P* < 0.001). For the “ESD marking” module, we detected slightly stronger negative correlations of completion time with years of endoscopy experience (Spearman’s r -0.26, *P* < 0.001), with number of EGDs performed (Spearman’s r -0.42, *P* < 0.001), and with the number of colonoscopies performed (Spearman’s r −0.40, *P* < 0.001; Figure S4). Taken together, the more experienced a participant was the faster was a module’s completion time.

## Discussion

This prospective study is the hitherto first validation of the novel Endonix® simulator that systematically accounted for all four qualities of validity. A large cohort of 310 delegates at two congresses with various backgrounds but similar gender distribution participated. Overall, the simulator was found to be feasible with good usability demonstrated by several items. The simulator was also found to be realistic reflecting the user’s individual experience. A majority of novice and beginner participants and a substantial proportion of experienced endoscopists, supported the integration of this simulator into structured endoscopy training.

The employment of two well-validated and widely used scores across multiple disciplines is another strength of this study; also given their yet infrequent use in simulator evaluation [17]. The SUS has been used across many industries and is one of the most popular, reliable and widely cited usability tools, valid and sensitive to a range of independent variables [29, 30]. The SUS regarding the Endonix® simulator revealed a median of 75 points, corresponding to good usability with a higher tendency in novices and beginners. To assess the individual task load of users, the validated and standardized NASA-TLX was also measured. NASA-TLX scores were significantly higher in novices compared to beginners or experienced endoscopists encompassing all six subdomains: mental, physical, and temporal demand, as well as performance, effort and frustration. The measured workload values in this study (i.e., the unweighted NASA-TLX), correspond to a level slightly below the median of other published workload intensities, as reported in a meta-analysis of over 200 studies [31]. As there is no universally accepted threshold defining high or low workload levels, the author of that meta-analysis recommends using relative comparisons between groups rather than absolute cut-offs to contextualize findings. Interestingly, frustration scores were slightly higher among experienced endoscopists than beginners, possibly reflecting the difference between the virtual environment and real endoscopic practice. Overall, the combined use of SUS and NASA-TLX yielded interesting results and may be considered in future simulator evaluations.

Participants were asked prior and after the simulation regarding their perception of the role of simulators in endoscopy training. 83.1% supported the use of any simulator in endoscopy training. Moreover, 61.9% agreed or strongly agreed to a mandatory simulator training prior to performing endoscopy in patients. However, only 57.3% of novices, 34.9% of beginners and 37.2% of experienced endoscopists found the Endonix® simulator to be realistic, which limits content validity, as this dimension should be based on expert assessments. The lower realism ratings among experts and beginners likely reflect their more nuanced understanding of real-world endoscopy, while novices may overestimate realism due to limited or lacking prior exposure. The low perceived realism also could be caused by the graphical representation in the simulation. Furthermore, the 3D-printed endoscopy handle gives the impression of a single-use endoscope rather than a reusable one. On the other hand, 74.4%, 62.2%, and 63.1% of participants expressed the desire to own the simulator at their institution, respectively. Most participants (54.1%) found the simulator to be useful for less-experienced endoscopists, indicating that novices and beginners might represent the most suitable target learners for this simulator.

Virtual simulators allow highly standardized and reproducible training. This enables objectification of a trainee’s performance and comparability of individual learning curves [32, 33]. Although most virtual simulators have limitations in the generation of a realistic environment, situations and anatomic structures, they do not require the maintenance or replacement of animal tissue and could be integrated into training software or interfaces for analysis and supervision of a trainee’s performance and thereby possibly reduce the necessary supervised training time [15, 34]. No single simulator meets all training requirements. The Endonix® simulator studied herein was evaluated and feasible to train tip control. A skill that is gaining attraction given the increased opportunities of endoscopic interventions that necessitate precise tip control. Such a simulation might not only be helpful for initial endoscopy training but also before starting interventions that require excellent tip control.

A previous virtual simulator (CAE EndoVR, CAE Healthcare, Montreal, Canada) distinguished the trained from the untrained and the resident from the expert. Although there were no significant differences between the senior residents and the experts, the expert commonly outperformed the residents [35]. On the other hand, comparative studies showed that residents who were simulator trained only showed an inferior performance compared to residents receiving a classical bedside teaching [36]. Randomized controlled trials (RCTs) showed poor content and criterion validity and failed to predict endoscopy skills during in vivo endoscopy and it was clearly shown using this simulator that constant feedback by a trainer/supervisor is necessary to increase a trainees’ learning curve [37]. Studies based on another VR simulator (ENDO Suite-GI Mentor, Simbionix Corp., Cleveland, USA later acquired by Surgical Science, Göteborg, Sweden) showed that a simulator training alone did not improve trainees’ skills but incorporation of a simulator in a structured training program was helpful [38]. In RCTs, the use of this VR simulator significantly affects technical accuracy in the early- and mid-term stages of endoscopic training and reduces the time needed to reach technical competency but could not replace on-patient training [39, 40]. Other virtual simulators (EndoSim, Surgical Science, Göteborg, Sweden; EndoVision Standard, Medvision / MSE Group, Vienna, Austria; and CLA 4/5, Coburger Lehrmittelanstalt, CLA, Coburg, Germany) have not been evaluated so far [17]. In summary, all these simulator evaluations either lacked prospective evaluation of all four validity criteria or did not include established evaluation tools such as the SUS or NASA-TLX.

This study has some limitations due to its design. While all delegates from two large congresses were invited to participate, some selection bias might have been introduced as this study relied on self-reported training and competency levels, which may be subject to bias due to self-assessment although plausibility checks were performed. Moreover, the evaluation of the usability or workload is inherently subjective and may be related to uncommon environment and stress levels during a congress. To overcome these potential limitations, standardized and well-established scores were used. To reduce stress levels, participation was offered during the whole congress days and participants had the opportunity to book a time slot that fits their personal schedule. Furthermore, given the exploratory nature of this study, no definitive conclusions can be drawn regarding the simulator’s impact on the learning curve of endoscopy trainees which warrants longitudinal examinations preferably in a randomized controlled trial comparing VR simulators to standard training without simulators.

In conclusion, the first prospective evaluation of the Endonix® simulator revealed it to be feasible mainly for novices and beginners. A robust content, face, construct and criterion validation assessment was achieved, posing it as a possible alternative simulator for endoscopy training. This study supports its potential role as a supplementary tool for structured endoscopy training, especially for beginners.

## Supplementary Information

Below is the link to the electronic supplementary material.

Supplementary file1 (MP4 172185 KB)

Supplementary file2 (MP4 60833 KB)

Supplementary file3 (TIF 11527 KB)

Supplementary file4 (TIF 34322 KB)

Supplementary file5 (TIF 14571 KB)

Supplementary file6 (TIF 39022 KB)

Supplementary file7 (TIF 41375 KB)

Supplementary file8 (DOCX 2714 KB)

## Data Availability

The data are available from the first and last authors upon reasonable request.

## Abbreviations

3D: Three-dimensional
CT: Computed tomography
DGVS: German Society for Gastroenterology, Digestive and Metabolic Diseases
ECN: ENDOCLUBNORD
ESD: Endoscopic submucosal dissection
ESGE: European Society of Gastrointestinal Endoscopy
JUGA: “Junge Gastroenterologie”-Young Gastroenterologist working group of the DGVS
NASA-TLX: National Aeronautics and Space Administration Task Load Index
RCT: Randomized controlled trial
SUS: System usability scale
VR: Virtual reality
