# Supplementary figures and images for: Feasibility, usability, and validity assessment of a novel plug-and-play virtual endoscopy simulator
Source: Surg Endosc. 2025 Dec 4;40(2):1629–40. doi: 10.1007/s00464-025-12396-8 (PMC12881084; doi:10.1007/s00464-025-12396-8)

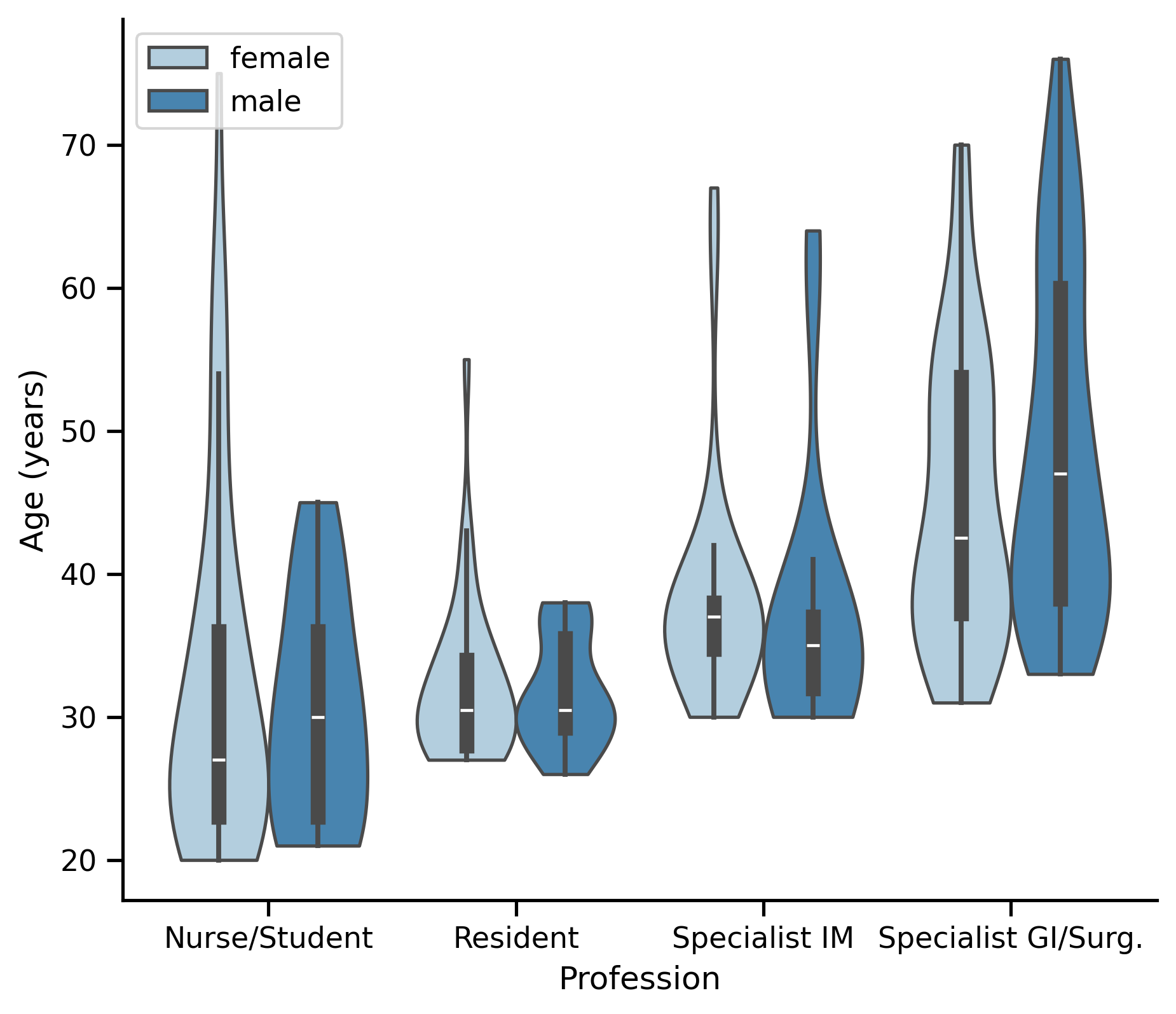

Supplement: Supplementary file 3 — Supplementary file3 (TIF 11527 KB) [file 464_2025_12396_MOESM3_ESM.tif]

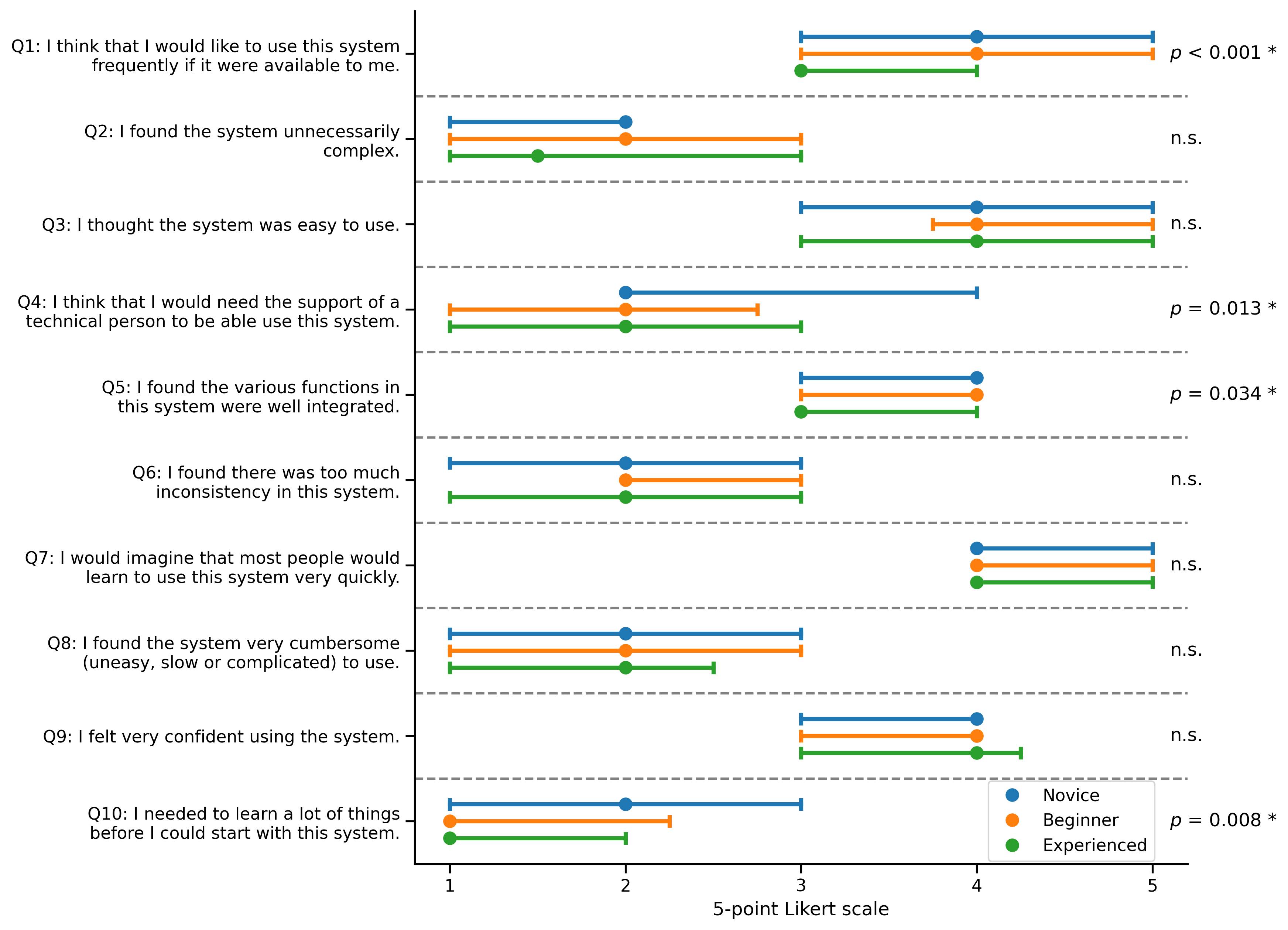

Supplement: Supplementary file 4 — Supplementary file4 (TIF 34322 KB) [file 464_2025_12396_MOESM4_ESM.tif]

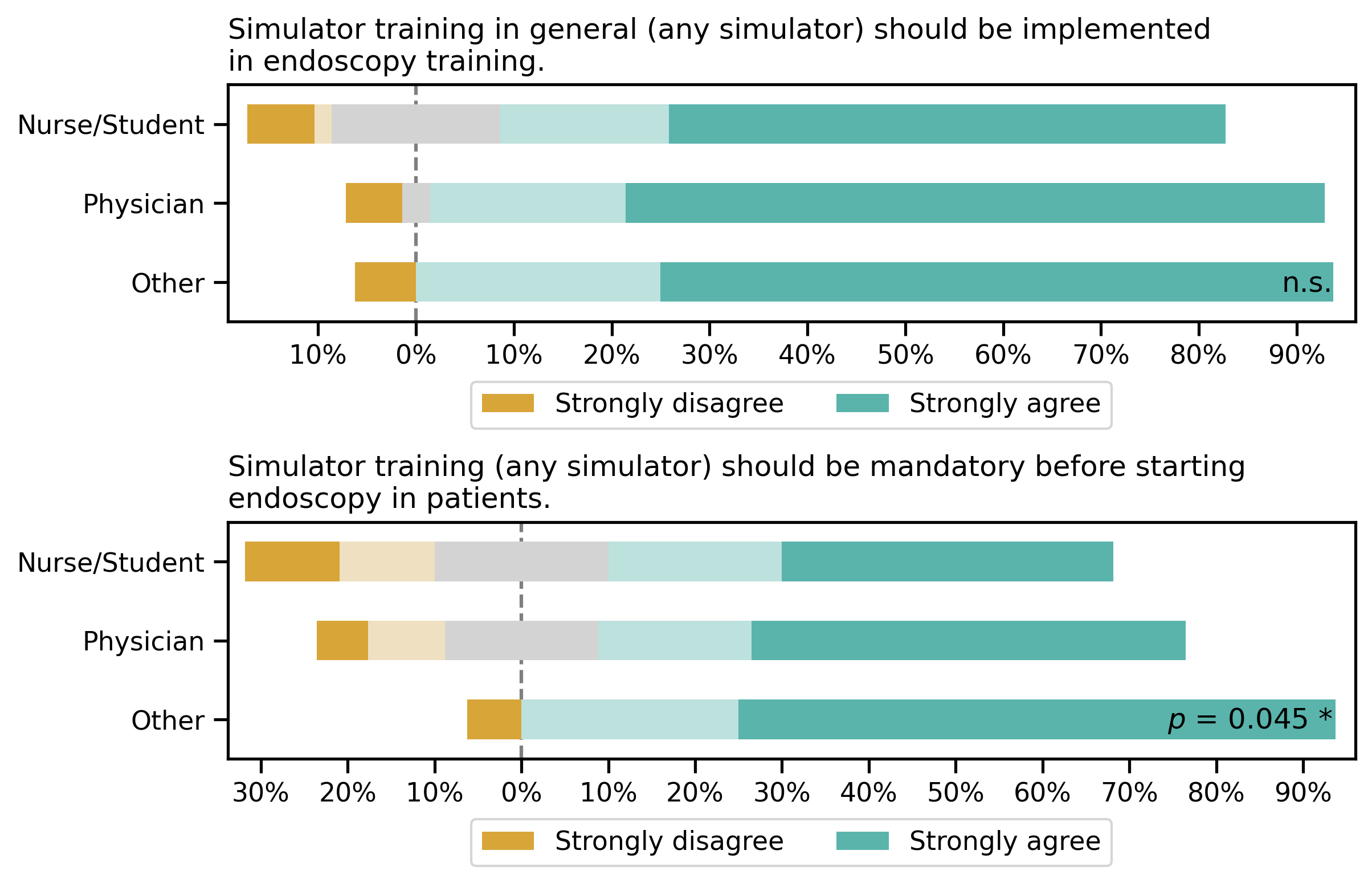

Supplement: Supplementary file 5 — Supplementary file5 (TIF 14571 KB) [file 464_2025_12396_MOESM5_ESM.tif]

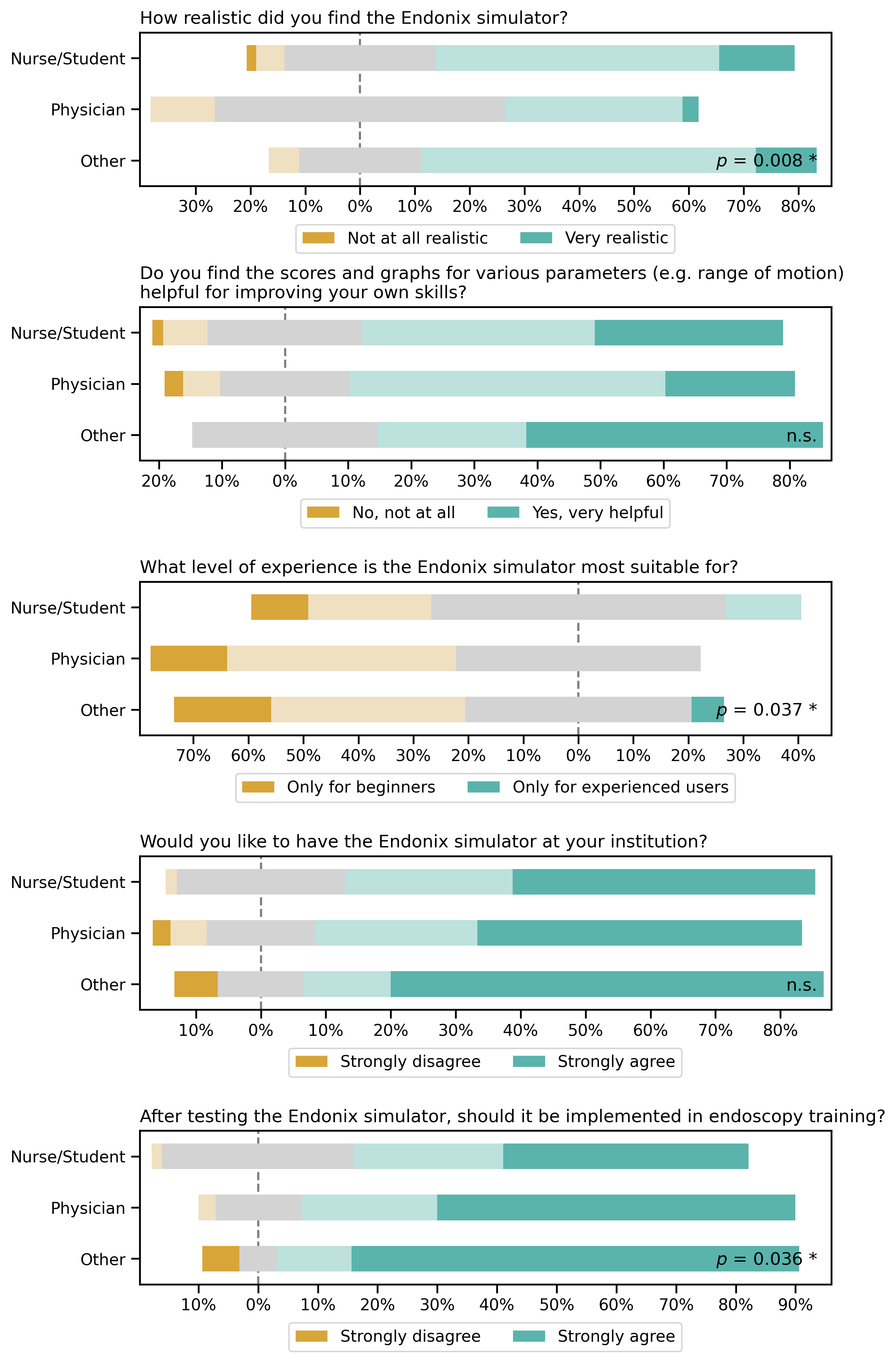

Supplement: Supplementary file 6 — Supplementary file6 (TIF 39022 KB) [file 464_2025_12396_MOESM6_ESM.tif]

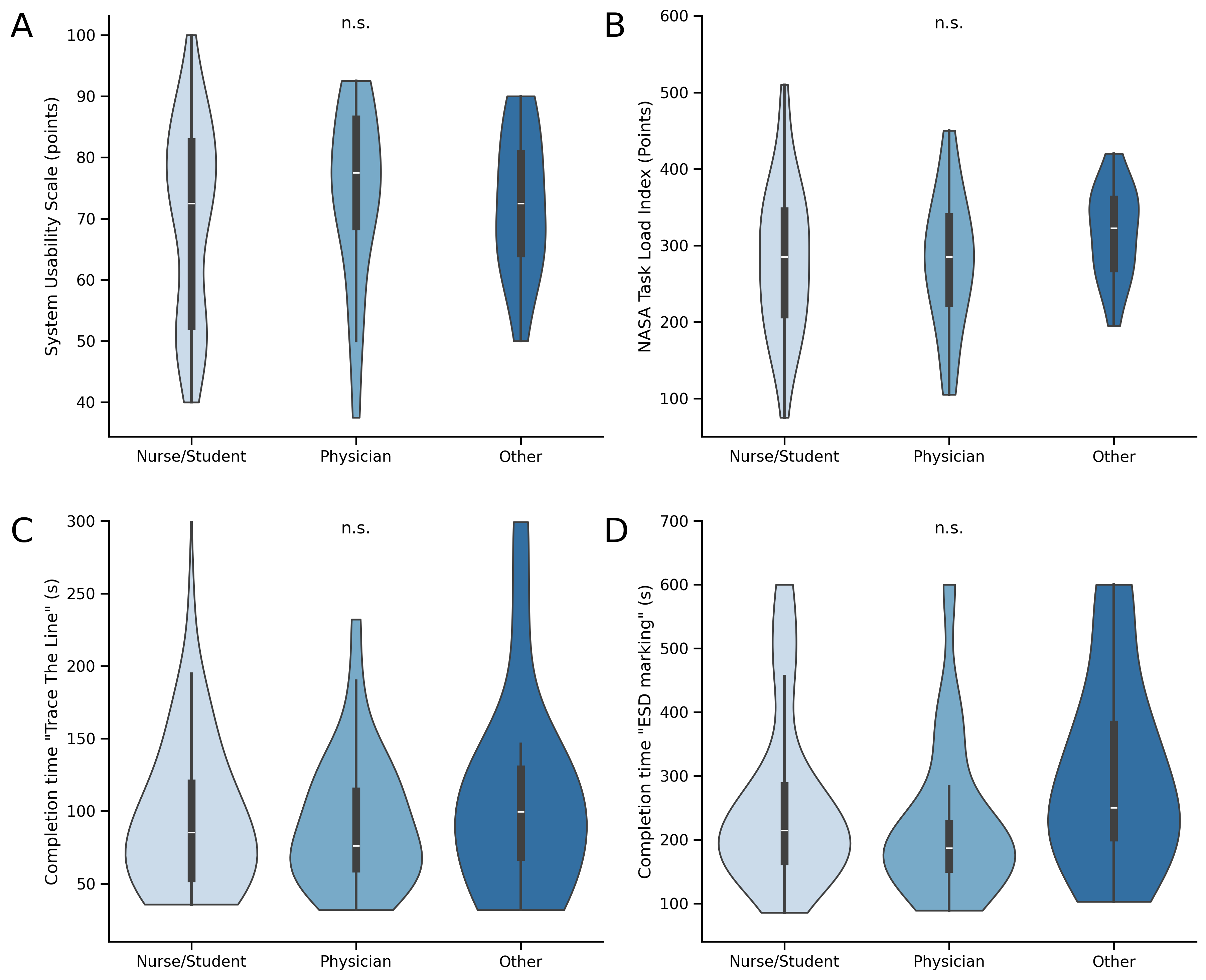

Supplement: Supplementary file 7 — Supplementary file7 (TIF 41375 KB) [file 464_2025_12396_MOESM7_ESM.tif]
