# Supplementary material for: Feasibility, usability, and validity assessment of a novel plug-and-play virtual endoscopy simulator
Source: Surg Endosc. 2025 Dec 4;40(2):1629–40. doi: 10.1007/s00464-025-12396-8 (PMC12881084; doi:10.1007/s00464-025-12396-8)
Supplement: Supplementary file 8 — Supplementary file8 (DOCX 2714 KB) [file 464_2025_12396_MOESM8_ESM.docx]

# Supplement of Hohlstein P et al.

**Feasibility, Usability, and Validity Assessment of a Novel Plug-and-play Virtual Endoscopy Simulator**

**Table of contents**

- **Supplementary Figures**
  - **Suppl. Figure S1.** Age distribution of study participants.
  - **Suppl. Figure S2.** Items of the System Usability Scale (SUS).
  - **Suppl. Figure S3.** Items of the NASA task load index (NASA TLX).
  - **Suppl. Figure S4.** Criterion validity of the virtual simulator.
  - **Suppl. Figure S5.** Perception of simulator training among different occupations backgrounds in novice endoscopists
  - **Suppl. Figure S6.** Content and face validity among different occupational backgrounds in novice endoscopists.
  - **Suppl. Figure S7.** Usability and construct validity among different occupational backgrounds in novice endoscopists.
- **Supplementary Videos**
  - **Suppl. Video 1.** Training module “Trace the Line”.
  - **Suppl. Video 2.** Training module “ESD marking”.
- **Suppl. Tables**
  - **Suppl. Table 1.** Baseline characteristics of novice endoscopists in comparison between different occupational backgrounds.

# Supplementary figures


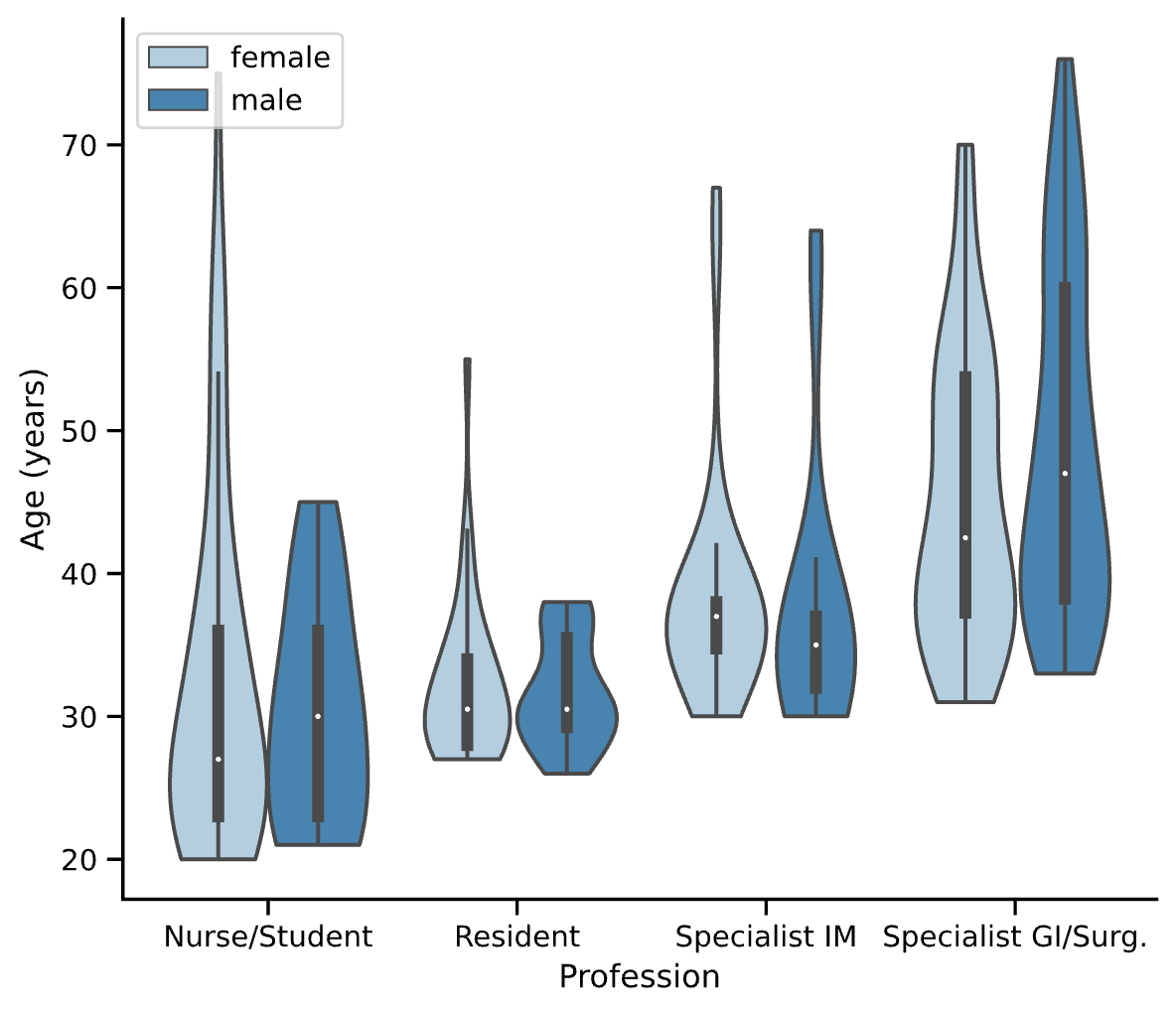


Suppl. Figure S1. Age distribution of study participants in comparison between the genders.


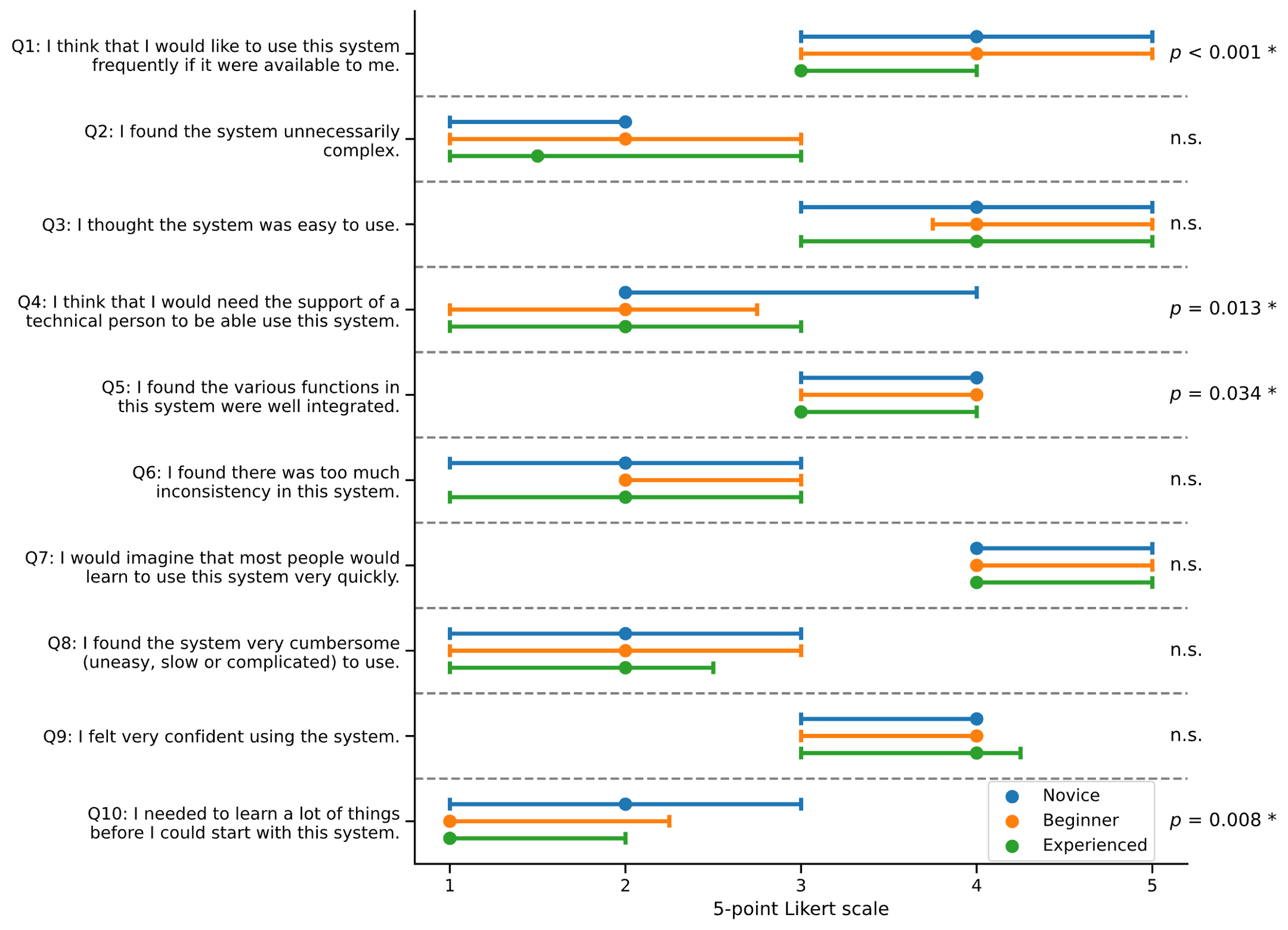
Suppl. Figure S2. Items of the System Usability Scale (SUS) on a Five-point Likert scale (1 = strongly disagree to 5 = strongly agree) in comparison between the groups. Dots represent the median value with surrounding whiskers indicating the interquartile range (IQR). * Significance between groups was assessed using the Kruskal Wallis test.


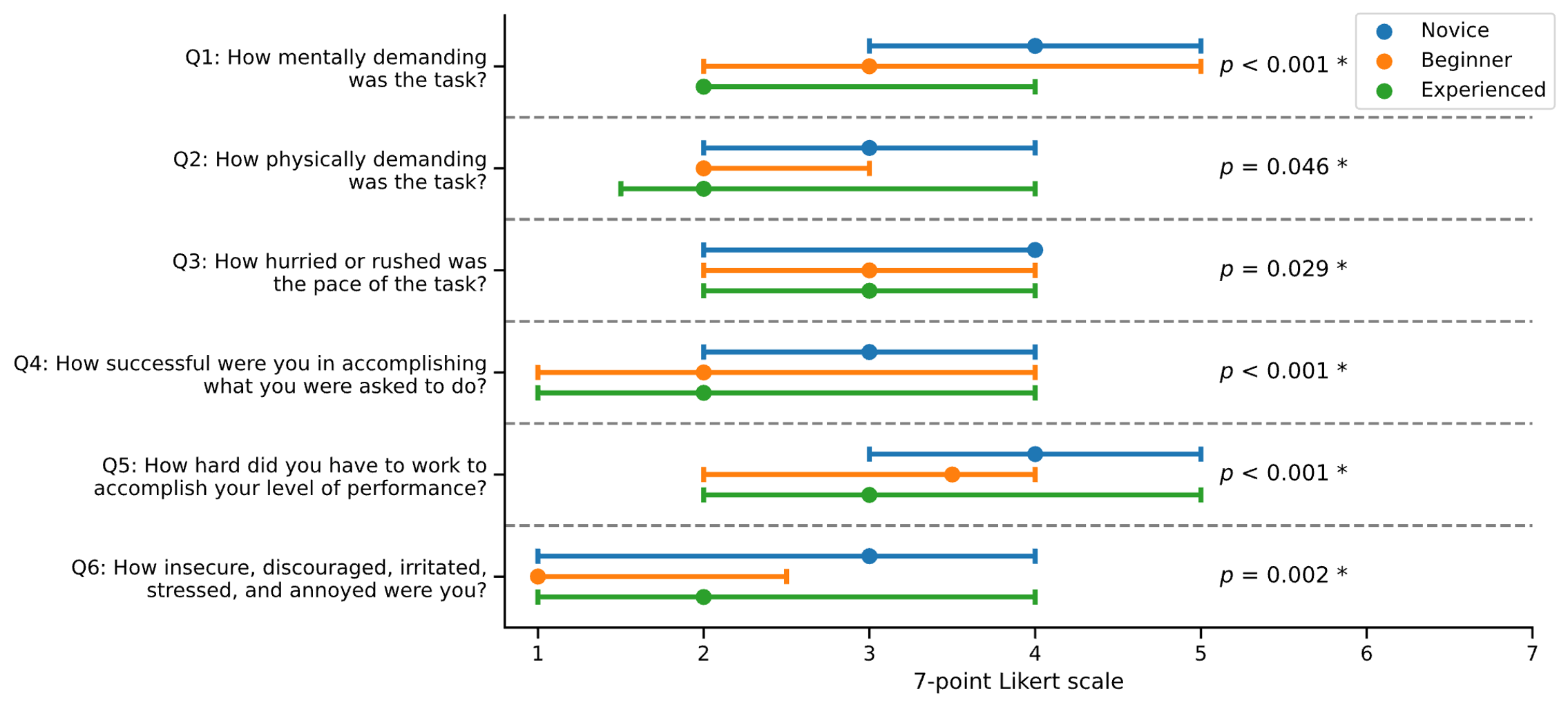
 Suppl. Figure S3. Items of the NASA task load index (NASA TLX) on a Seven-point Likert scale (1 = very low to 7 = very high, except Q4 1 = perfect to 7 = failure) in comparison between the groups. Dots represent the median value with surrounding whiskers indicating the interquartile range (IQR). * Significance between groups was assessed using the Kruskal Wallis test.


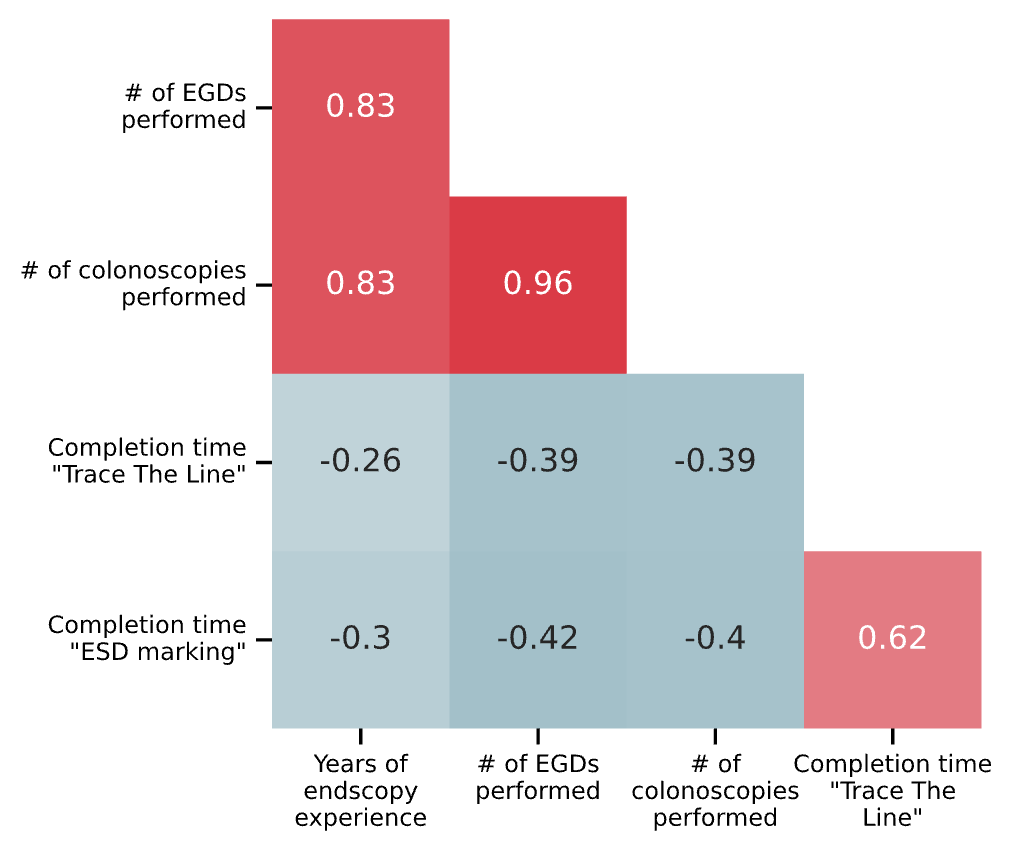


Suppl. Figure S4. Criterion validity of the virtual simulator. Spearman correlation coefficients between completion times of the modules „Trace The Line“ and „ESD marking“ and measures of endoscopic experience. All *p*-values < 0.001.


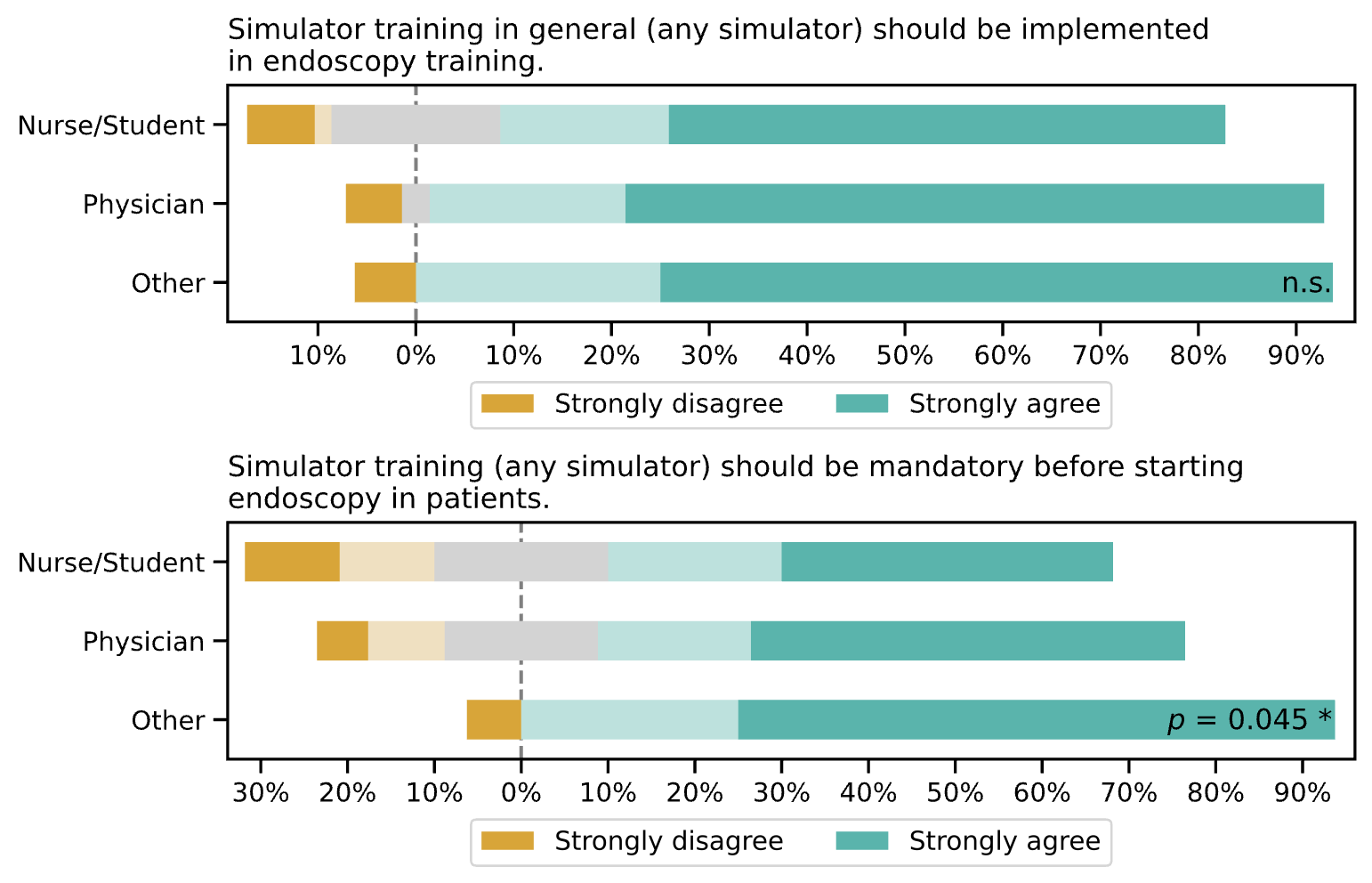


Suppl. Figure S5. Perception of simulator training in endoscopy among different occupational backgrounds in novices (Nurse / Student, Physician, Other), assessed using 5-point Likert scales (1 = strongly disagree, 5 = strongly agree). Top: General support for implementing simulator training in endoscopy curricula. Bottom: Opinion on mandatory simulator training prior to performing endoscopy on patients. Using the Kruskal Wallis test, no significant differences between the groups were detected for both questions.


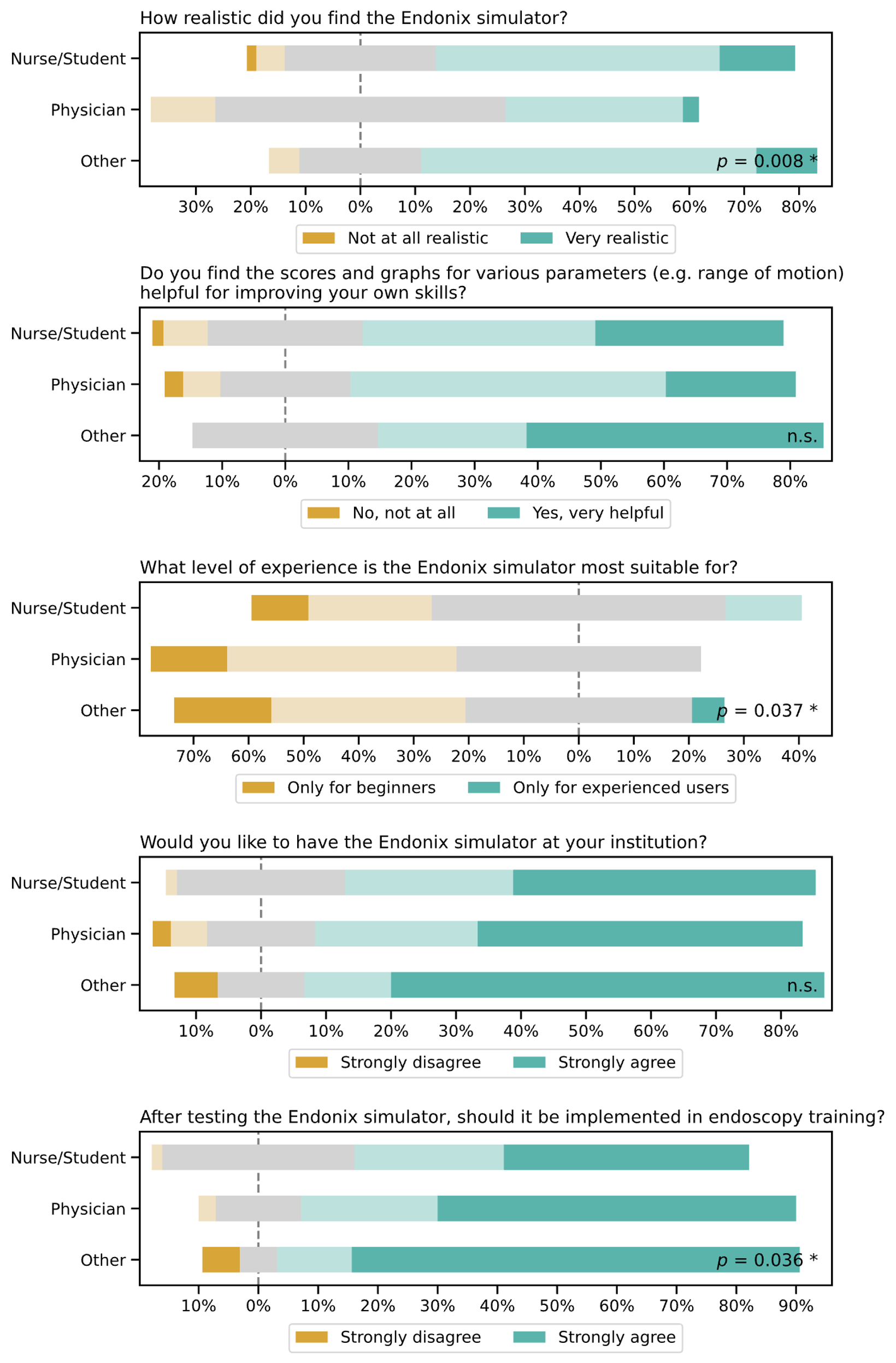


Suppl. Figure S6. Evaluation of content and face validity of the virtual Endonix simulator on a Five-point Likert scales (description below plots) assessing realism, usefulness of feedback metrics, target group, interest in institutional availability, and integration into training depending on occupational background among novices (Nurse / Student, Physician, Other). *Significance (p < 0.05) between groups was assessed using the Kruskal-Wallis test.


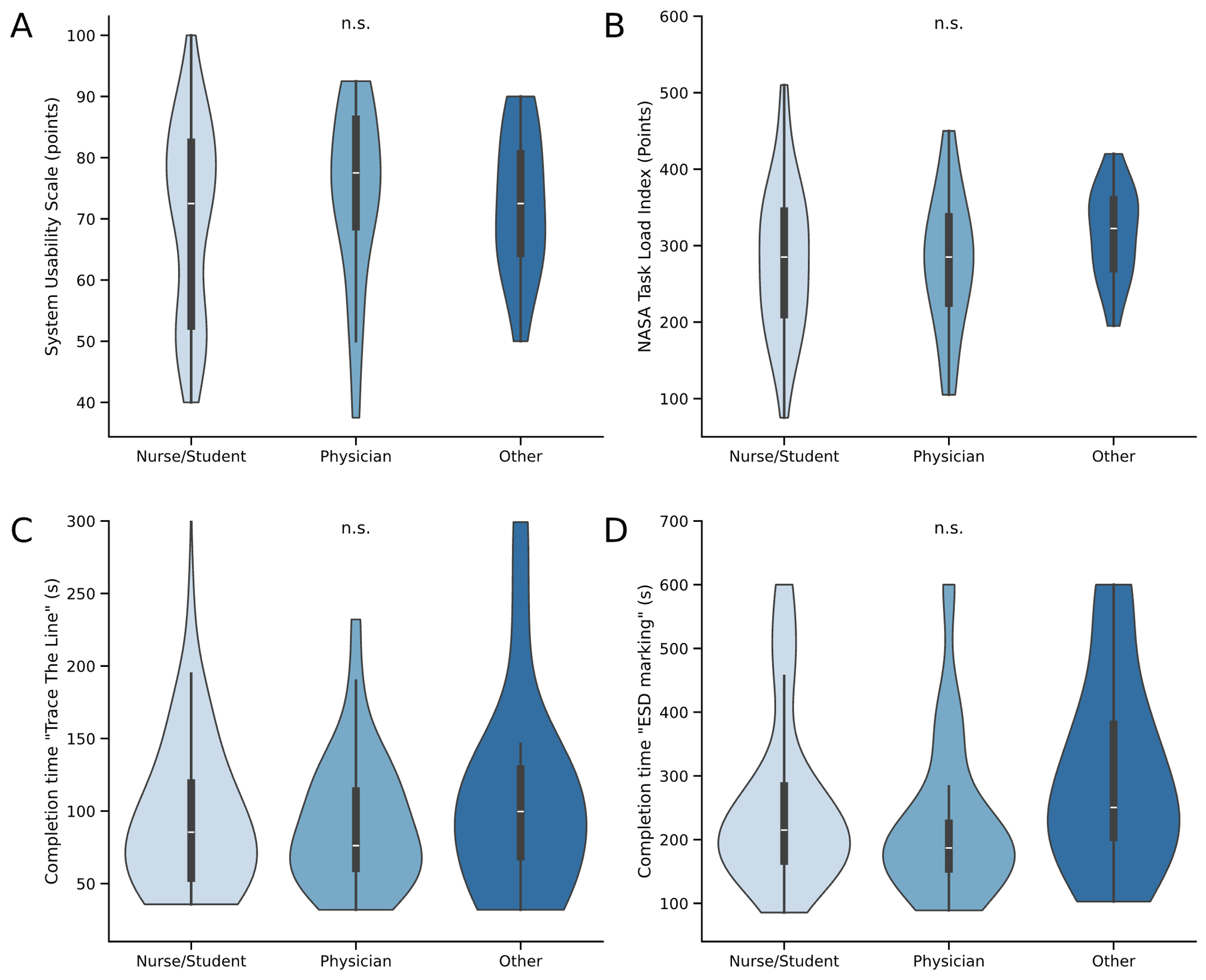


Suppl. Figure S7. Usability and construct validity of the virtual Endonix simulator among different occupational backgrounds in novices. (A) System usability scale (SUS) and (B) NASA Task Load Index (NASA TLX) across endoscopy experience levels. Time to completion of the exercise „Trace the Line“ (C) and „ESD marking (D). *Significance (P <0.05) between groups was assessed using the Kruskal-Wallis test, followed by Dunn’s multiple comparison test.

# Supplementary videos

FILE: Endonix_TTL.mp4

Suppl. Video 1. Training module “Trace the Line”.

FILE: Endonix_EM.mp4

Suppl. Video 2. Training module “ESD marking”.

# Supplementary tables

**Suppl. Table S1.** Baseline characteristics of novice endoscopists in comparison between different occupational backgrounds.

|  | Nurse / Student | Physician | Other |
| --- | --- | --- | --- |
| n | 60 | 36 | 18 |
| Congress (DGVS / ENDOCLUBNORD) | 27 / 33 | 32 / 4 | 7 / 11 |
| Age, years (IQR) | 27 (12.25) | 30.5 (4.75) | 35 (5.5) |
| Female gender, n (%) | 46 (76.7) | 21 (58.3) | 12 (66.7) |
| Profession (Nurse or Student / Resident / Specialist IM / Specialist GI or Surgery / Other), n (%) | 60 / 0 / 0 / 0 / 0  (100 / 0 / 0 / 0 / 0) | 0 / 29 / 1 / 6 / 0  (0 / 80.6 / 2.8 / 16.7 / 0) | 0 / 0 / 0 / 0 / 18  (0 / 0 / 0 / 0 / 100) |
| Country of current practice (DE or AT or CH / Other EU / Non EU or missing), n (%) | 54 / 3 / 3  (90.0 / 5.0 / 5.0) | 36 / 0 / 0  (100 / 0 / 0) | 17 / 1 / 0  (94.4 / 5.6 / 0) |
| Institution owns simulator, n (%) | 11 (18.3) | 6 (16.7) | 3 (16.7) |
| Institution with structured curriculum, n (%) | 10 (16.7) | 9 (25.0) | 32 (11.1) |
| Completed module „Trace the Line“, n (%) | 59 (98.3) | 36 (100) | 18 (100) |
| Completed module „ESD marking“, n (%) | 54 (90.0) | 34 (94.4) | 16 (88.9) |

The median and interquartile range (IQR, in parentheses) are given unless indicated otherwise. Abbreviations: GI: gastrointestinal; EGD: esophagogastroduodenoscopy; ESD: endoscopic submucosal dissection.
